# Efficacy and safety of gemcitabine plus S-1 *vs.* gemcitabine plus nab-paclitaxel in treatment-naïve advanced pancreatic ductal adenocarcinoma

**DOI:** 10.20892/j.issn.2095-3941.2023.0189

**Published:** 2023-08-29

**Authors:** Zhou Zhu, Hui Tang, Jinrong Ying, Yuejuan Cheng, Xiang Wang, Yingyi Wang, Chunmei Bai

**Affiliations:** 1 Department of Medical Oncology, Peking Union Medical College Hospital, Chinese Academy of Medical Sciences & Peking Union Medical College, Beijing 100730, China; 2 4+4 Medical Doctor Program, Chinese Academy of Medical Sciences & Peking Union Medical College, Beijing 100730, China

**Keywords:** Advanced pancreatic cancer, first-line chemotherapy, gemcitabine, S-1, nab-paclitaxel

## Abstract

**Objective:**

Gemcitabine plus nab-paclitaxel (GnP) is the standard first-line therapy for advanced pancreatic ductal adenocarcinoma (PDAC). S-1, an oral fluoropyrimidine derivative, as compared with gemcitabine, is non-inferior in terms of overall survival (OS) and is associated with lower hematologic toxicity. Accordingly, S-1 is a convenient oral alternative treatment for advanced PDAC. This study was aimed at comparing the efficacy and safety of gemcitabine plus S-1 (GS) *vs.* GnP as first-line chemotherapy for advanced PDAC.

**Methods:**

Patients with advanced PDAC who received first-line GS or GnP at the Peking Union Medical College Hospital between March 2011 and November 2022 were evaluated.

**Results:**

A total of 300 patients were assessed, of whom 84 received GS and 216 received GnP. The chemotherapy completion rate was higher with GS than GnP (50.0% *vs.* 30.3%, *P* = 0.0028). The objective response rate (ORR) was slightly higher (14.3% *vs.* 9.7%, *P* = 0.35), and the median OS was significantly longer (17.9 months *vs.* 13.3 months, *P* = 0.0078), in the GS group than the GnP group. However, the median progression-free survival (PFS) did not significantly differ between groups. Leukopenia risk was significantly lower in the GS group than the GnP group (14.9% *vs.* 28.1%, *P* = 0.049).

**Conclusions:**

As first-line chemotherapy for advanced PDAC, the GS regimen led to a significantly longer OS than the GnP regimen. The PFS, ORR, and incidence of severe adverse events were comparable between the GS and GnP groups.

## Introduction

Pancreatic ductal adenocarcinoma (PDAC) is among the most aggressive and deadly diseases, and has a poor prognosis. PDAC is currently the fourth leading cause of cancer-related deaths and is expected to become the second leading cause by 2030^1^. Only 20% of patients with PDAC can be initially treated with surgical resection. Therefore, systemic chemotherapy is central in the treatment of advanced PDAC. Despite modest progress in gemcitabine-based chemotherapy, the 5-year survival rate for distant PDAC is as low as 3%^2^.

Because gemcitabine has been reported to provide a modest advantage over 5-fluorouracil (5-FU) in terms of median overall survival (OS) (5.65 *vs.* 4.41 months) in patients with advanced PDAC^3^, many trials have explored more effective combination regimens based on gemcitabine or 5-FU as first-line chemotherapy. In the PRODIGE 4/ACCORD 11 trial, a chemotherapy regimen consisting of fluorouracil, leucovorin, irinotecan, and oxaliplatin (FOLFIRINOX) achieved significant improvements over gemcitabine monotherapy in terms of OS (11.1 *vs.* 6.8 months) and progression-free survival (PFS) (6.4 *vs.* 3.3 months) in patients with metastatic PDAC with good performance status^4^. In 2013, the MPACT trial, comparing gemcitabine plus nab-paclitaxel (GnP) *vs.* gemcitabine alone in patients with metastatic pancreatic cancer, found significantly superior OS (8.5 *vs.* 6.7 months) and PFS (5.5 *vs.* 3.7 months) with GnP^5^. However, the objective response rate (ORR) with GnP (23%) or FOLFIRINOX (31.6%) was limited, and the OS was less than 1 year. Both GnP and FOLFIRINOX were associated with increases in grade 3 or higher myelosuppression, and peripheral neuropathy. Hence, the development of more effective and tolerant combination regimens remains a major unmet need for patients with advanced PDAC.

S-1 is an oral fluoropyrimidine derivative comprising tegafur, gimeracil, and oteracil potassium in a molar ratio of 1.0:0.4:1.0^6^. In a randomized phase III study, S-1 exhibited non-inferiority to gemcitabine, in terms of OS and lower hematologic toxicity, in patients with advanced PDAC^7^. Preclinical studies have revealed synergistic cytotoxic effects of gemcitabine combined with S-1 in murine models^8,9^. To date, 4 randomized controlled trials (RCTs) have directly compared gemcitabine plus S-1 (GS) with gemcitabine monotherapy as first-line chemotherapy for patients with advanced PDAC^7,10–12^. These RCTs have indicated that GS significantly improves PFS and ORR, and is associated with acceptable treatment-associated toxicity. However, whether the combined therapy improved OS was inconsistent among these trials. In addition, no head-to-head comparisons of GS with other combined regimens used as first-line chemotherapy have been performed.

In this study, we compared the efficacy and safety of GS and GnP as first-line chemotherapy for patients with advanced PDAC.

## Materials and methods

### Patients

This study retrospectively included patients administered GS or GnP as first-line chemotherapy between March 18, 2011, and November 29, 2022, at the Peking Union Medical College Hospital (Beijing, China). Patient data were retrieved from the Electronic Medical Record Analytical Database (PUMCH-EMERALD).

The inclusion criteria were as follows: (1) patients with histologically or cytologically confirmed locally advanced or metastatic PDAC, (2) patients older than 18 years, (3) patients administered at least one cycle of GS or GnP as first-line chemotherapy, and (4) patients with adequate hematologic, hepatic, and renal function. The exclusion criteria were as follows: (1) patients who died or were lost to follow-up before the first efficacy assessment, (2) patients with other primary tumors, and (3) patients who had received systemic antitumor therapy.

Ethical approval was obtained from the Medical Ethics Committee of Peking Union Medical College Hospital (Approval No. S-K2099). This study conformed to the principles of the Declaration of Helsinki and the ethical requirements for studies involving human participants. Informed consent was not required, because of the retrospective nature and anonymization of the study data.

### Treatment and assessment

In the GS group, patients were intravenously administered gemcitabine (1,000 mg/m^2^) on days 1 and 8 plus oral S-1 [40 mg for body-surface area (BSA) < 1.25 m^2^, 50 mg for BSA ≥ 1.25 m^2^ but < 1.5 m^2^, or 60 mg for BSA ≥ 1.5 m^2^] twice per day for 14 days, followed by a 7-day pause (1 cycle). Patients in the GnP group received intravenous nab-paclitaxel (125 mg/m^2^) and gemcitabine (1,000 mg/m^2^) on days 1, 8, and 15, every 4 weeks. Treatment continued until the occurrence of intolerable toxicity, disease progression, or discontinuation at the discretion of the investigators or patients. The chemotherapy completion rate was defined as the proportion of patients who completed 6 months of therapy and an appropriate number of chemotherapy cycles.

The primary endpoint was OS, defined as the interval between the initiation of first-line chemotherapy and death from any cause. The secondary endpoints were PFS, ORR, and safety. PFS was defined as the time from the initiation of chemotherapy to disease progression or death from any cause. Contrast-enhanced computed tomography or magnetic resonance imaging was performed at baseline and every 2–3 months after chemotherapy initiation to assess tumor response according to Response Evaluation Criteria in Solid Tumors (RECIST), version 1.1^13^. Carbohydrate antigen 19-9 (CA19-9) levels were measured at baseline and then every 2–3 months. Treatment-associated adverse events (AEs) were evaluated according to the National Cancer Institute Common Terminology Criteria for Adverse Events (CTCAE), version 5.0. All patients were followed up until death or loss of contact; the last day of follow-up was March 6, 2023.

### Statistical analysis

All statistical analyses were performed in R version 4.2.2 (https://www.r-project.org/). The Mann-Whitney U test or Pearson’s chi-square test was conducted to compare continuous or categorical variables. The Cox proportional hazards model was used to calculate the hazard ratios (HRs) with 95% confidence intervals (CIs) of variables associated with patient OS and PFS. The proportional hazard assumptions for the Cox regression models used in this study were tested with Schoenfeld residuals. No substantial deviations from the proportional hazard assumptions were observed. Survival curves were generated *via* Kaplan-Meier analyses and compared with the log-rank test. Propensity score matching (PSM) was used to minimize the effects of confounding factors. The propensity scores were calculated on the basis of age, gender, ECOG performance status score, baseline stage, baseline CA19-9 level, surgery before chemotherapy, and liver metastases. A 2-tailed probability value of *P* < 0.05 was considered to indicate statistical significance.

## Results

### Patient characteristics

A total of 300 patients with locally advanced (37.0%) or metastatic (63.0%) PDAC were evaluated according to the selection criteria. The baseline characteristics of the study cohort are presented in **Table 1**. Among the patients, 84 received GS and 216 received GnP as first-line chemotherapy. The median age was 61 years (range, 32–83), and 61.0% (*n* = 183) of the patients were men. Most patients had ECOG performance status scores of 0 (52.0%) or 1 (46.3%). Furthermore, patients treated with GS were generally older [median (range) age: 63 (38–83) *vs.* 60 (32–77) years; *P* = 0.017] than those treated with GnP; however, the chemotherapy completion rate was significantly higher among patients administered GS than GnP (50.0% *vs.* 30.3%, *P* = 0.0028). A cohort of 142 patients was generated with PSM, of whom 71 were treated with GS and 71 were treated with GnP. The clinical profiles of the matched patients are shown in **Supplementary Table S1**.

**Table 1:** Baseline characteristics of the 300 patients

Characteristics	Overall (*n* = 300)	First-line chemotherapy regimen		*P* value
		GS (*n* = 84)	GnP (*n* = 216)	
Gender				0.324
Male	183 (61.0%)	47 (56.0%)	136 (63.0%)	
Female	117 (39.0%)	37 (44.0%)	80 (37.0%)	
Age, median (range), years	61 (32, 83)	63 (38, 83)	60 (32, 77)	0.017
ECOG				0.112
0	156 (52.0%)	48 (57.1%)	108 (50.0%)	
1	139 (46.3%)	33 (39.3%)	106 (49.1%)	
2	5 (1.7%)	3 (3.6%)	2 (0.9%)	
BMI, median (range), kg/m^2^	21.6 (15.0, 35.3)	21.6 (15.0, 27.6)	21.5 (15.6, 35.3)	0.978
Smoking history				0.081
Yes	122 (40.7%)	27 (32.1%)	95 (44.0%)	
No	178 (59.3%)	57 (67.9%)	121 (56.0%)	
Drinking history				0.121
Yes	70 (23.3%)	14 (16.7%)	56 (25.9%)	
No	230 (76.7%)	70 (83.3%)	160 (74.1%)	
Diabetes history				1.000
Yes	92 (30.7%)	26 (31.0%)	66 (30.6%)	
No	208 (69.3%)	58 (69.0%)	150 (69.4%)	
Surgery before chemotherapy				0.742
Yes	48 (16.0%)	12 (14.3%)	36 (16.7%)	
No	252 (84.0%)	72 (85.7%)	180 (83.3%)	
CA19-9, median (range), U/mL	418 (0, 400,000)	343 (0, 400,000)	478 (0.3, 86,900)	0.300
Baseline stage				0.492
Locally advanced	111 (37.0%)	28 (33.3%)	83 (38.4%)	
Metastatic	189 (63.0%)	56 (66.7%)	133 (61.6%)	
Liver metastases				0.738
Yes	140 (46.7%)	41 (48.8%)	99 (45.8%)	
No	160 (53.3%)	43 (51.2%)	117 (54.2%)	
Multiple metastases				0.347
Yes	47 (15.7%)	10 (11.9%)	37 (17.1%)	
No	253 (84.3%)	74 (88.1%)	179 (82.9%)	

### Efficacy

The responses to GS and GnP treatment are shown in **Table 2**. Among all patients, 33 (11.0%) had partial response, 196 (65.3%) had stable disease, and 71 (23.7%) had progressive disease, according to RECIST1.1. No patients achieved complete response in the 2 groups. The ORR was slightly higher in the GS group than the GnP group (14.3% *vs.* 9.7%, *P* = 0.353). The disease control rates (DCRs) were similar between groups (81.0% *vs.* 74.5%, *P* = 0.307).

**Table 2:** Response to chemotherapy among the 300 patients

Items	Overall (*n* = 300)	First-line chemotherapy regimen		*P* value
		GS (*n* = 84)	GnP (*n* = 216)	
Best response				0.328
CR	0	0	0	
PR	33 (11.0%)	12 (14.3%)	21 (9.7%)	
SD	196 (65.3%)	56 (66.7%)	140 (64.8%)	
PD	71 (23.7%)	16 (19.0%)	55 (25.5%)	
ORR	11.0%	14.3%	9.7%	0.353
DCR	76.3%	81.0%	74.5%	0.307
Change in CA19-9				0.104
Not expressed	53 (17.7%)	20 (23.8%)	33 (15.3%)	
Declined > 30%	162 (54.0%)	47 (56.0%)	115 (53.2%)	
Declined ≤ 30%	67 (22.3%)	15 (17.9%)	52 (24.1%)	
Unknown	18 (6.0%)	2 (2.4%)	16 (7.4%)	

CR, complete response; DCR, disease control rate; ORR, objective response rate; PD, progressive disease; PR, partial response; SD, stable disease.

Of the 229 patients with elevated CA19-9 levels at baseline, the levels in 53 patients (23.1%) decreased to the normal range after GS or GnP treatment. Furthermore, 75.8% (47/62) of the GS group and 68.9% (115/167) of the GnP group achieved the greatest decrease (> 30%). After minimization of the influence of confounding factors with PSM, the best responses, ORR, DCR, and CA19-9 levels were comparable between groups (**Supplementary Table S2**).

### Survival

**Figure 1:**
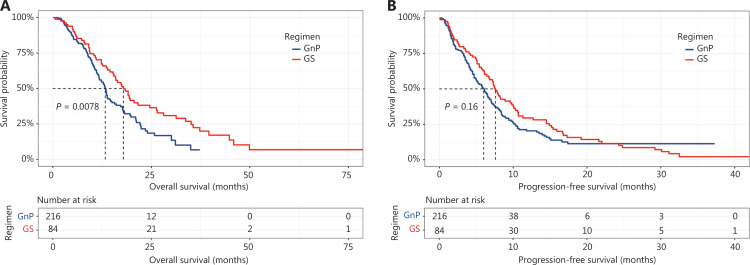
Kaplan-Meier curves of overall survival (A) and progression-free survival (B) for all patients.

**Figure 2:**
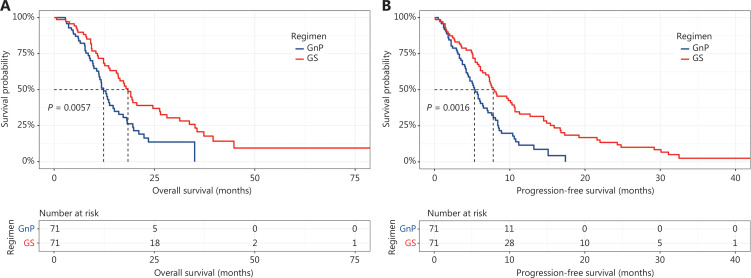
Kaplan-Meier curves of overall survival (A) and progression-free survival (B) for matched patients.

The median follow-up time was 19.8 months (range, 0.6–145.7). The median OS was significantly longer in the GS group than the GnP group [17.9 months (95% CI, 15.3–26.3 months) *vs.* 13.3 months (95% CI, 11.8–15.0 months); *P* = 0.0078] (**Figure 1A**). However, the median PFS [7.6 months (95% CI, 6.4–10.0 months) *vs.* 6.1 months (95% CI, 5.0–7.0 months), *P* = 0.16] did not differ significantly between the GS and GnP groups (**Figure 1B**). Nevertheless, the median PFS [7.8 months (95% CI, 6.8–10.7 months) *vs.* 5.4 months (95% CI, 4.5–7.1 months), *P* = 0.0016] significantly differed in the matched cohort (**Figure 2B**). The median OS remained longer for patients treated with GS than GnP [18.4 months (95% CI, 15.9–26.5 months) *vs.* 12.9 months (95% CI, 11.2–17.2 months); *P* = 0.0057] (**Figure 2A**).

**Figure 3:**
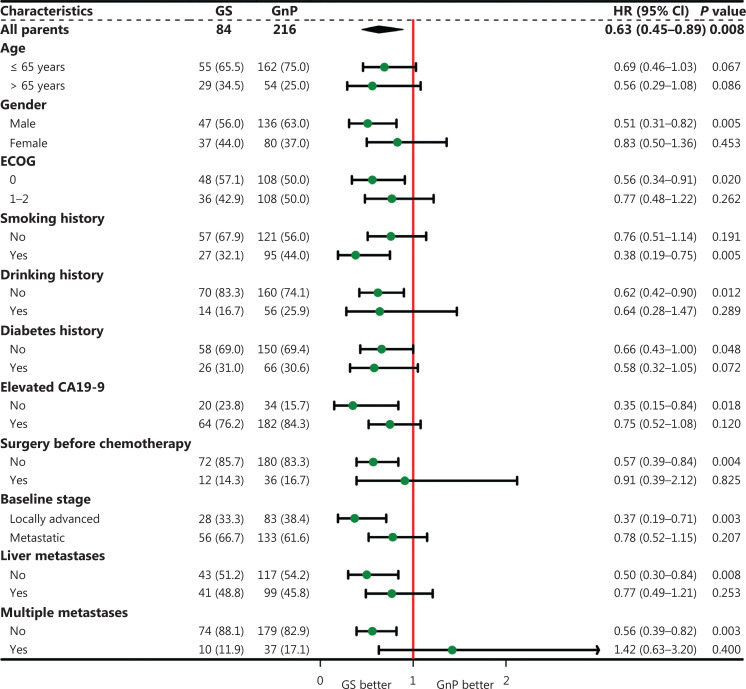
Forest plot of overall survival in the selected subgroups.

**Figure 4:**
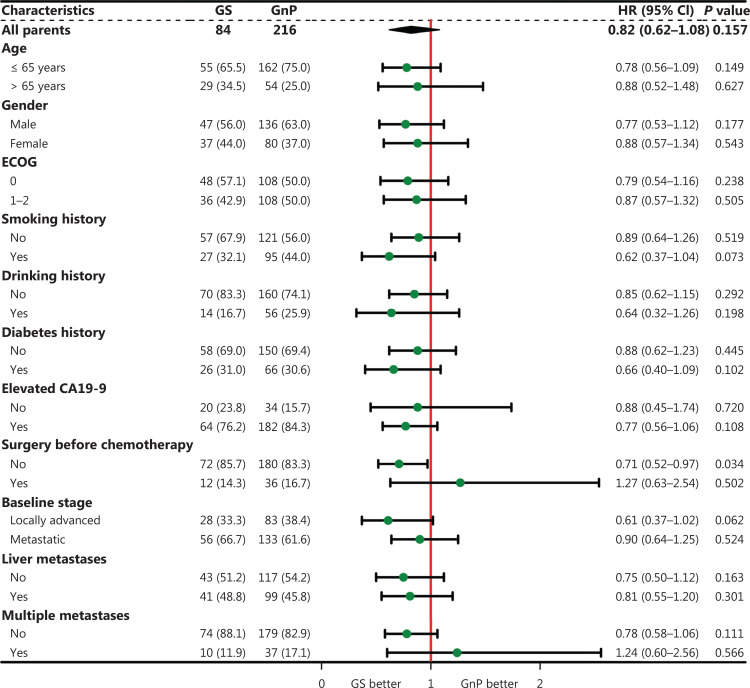
Forest plot of progression-free survival in the selected subgroups.

Subgroup analyses revealed that patients receiving GS as first-line chemotherapy achieved longer OS than those receiving GnP [hazard ratio (HR), 0.63; 95% CI, 0.45–0.89; *P* = 0.008], particularly male patients (HR, 0.51; 95% CI, 0.31–0.82; *P* = 0.005); patients with a smoking history (HR, 0.38; 95% CI, 0.19–0.75; *P* = 0.005); patients with an ECOG performance status score of 0 (HR, 0.56; 95% CI, 0.34–0.91; *P* = 0.020); patients with localized PDAC (HR, 0.37; 95% CI, 0.19–0.71; *P* = 0.003); and patients without elevated CA19-9 (HR, 0.35; 95% CI, 0.15–0.84; *P* = 0.018), liver metastases (HR, 0.50; 95% CI, 0.30–0.84; *P* = 0.008), or multiple metastases (HR, 0.56; 95% CI, 0.39–0.82; *P* = 0.003) (**Figure 3**). However, PFS was comparable between the GS and GnP groups in almost all subgroups (**Figure 4**).

The results of univariate and multivariate Cox analyses, performed to evaluate the potential prognostic factors associated with OS and PFS for all 300 patients, are presented in **Tables 3 and 4**, respectively. Univariate analysis revealed that ECOG performance status, baseline stage, baseline CA19-9 level, liver metastases, multiple metastases, and first-line chemotherapy regimens were significantly associated with OS. Furthermore, multivariate analysis confirmed that poor ECOG performance status, high CA19-9 levels before chemotherapy, liver metastases, and first-line GnP regimen were independent risk factors for poor OS (**Table 3**). ECOG performance status and liver metastases were also independently associated with PFS in the multivariate analysis. No first-line chemotherapy regimen (GS or GnP) was selected (**Table 4**). However, the choice of chemotherapy regimen was significantly associated with both OS and PFS in the propensity-matched cohort (**Supplementary Tables S3 and S4**).

**Table 3:** Univariate and multivariate analyses of overall survival

Variables	Univariate analysis		Multivariate analysis	
	HR (95% CI)	*P* value	HR (95% CI)	*P* value
Age, years	0.99 (0.97–1.01)	0.297	-	-
Gender (male *vs.* female)	1.01 (0.75–1.38)	0.925	-	-
ECOG (1 *vs.* 0)	1.85 (1.37–2.51)	**< 0.001**	1.93 (1.40–2.64)	**< 0.001**
ECOG (2 *vs.* 0)	0.59 (0.14–2.40)	0.459	0.71 (0.17–2.92)	0.636
BMI, kg/m^2^	0.98 (0.93–1.03)	0.433	-	-
Smoking history (yes *vs.* no)	0.96 (0.70–1.30)	0.771	-	-
Drinking history (yes *vs.* no)	0.85 (0.59–1.22)	0.380	-	-
Diabetes history (yes *vs.* no)	1.06 (0.77–1.46)	0.718	-	-
Surgery before chemotherapy (yes *vs.* no)	0.90 (0.59–1.36)	0.605	-	-
Baseline stage (metastatic *vs.* locally advanced)	1.70 (1.23–2.34)	**0.001**	0.94 (0.57–1.53)	0.789
Baseline CA19-9 level, U/mL	1.00 (1.00–1.00)	**< 0.001**	1.00 (1.00–1.00)	**< 0.001**
Liver metastases (yes *vs.* no)	1.93 (1.43–2.62)	**< 0.001**	2.12 (1.34–3.34)	**0.001**
Multiple metastases (yes *vs.* no)	1.57 (1.05–2.33)	**0.026**	1.20 (0.78–1.84)	0.419
First-line regimen (GS *vs.* GnP)	0.63 (0.45–0.89)	**0.008**	0.69 (0.48–0.97)	**0.035**

The bold values indicate *P* < 0.1 in the univariable analysis and *P* < 0.05 in the multivariable analysis.

**Table 4:** Univariate and multivariate analyses of progression-free survival

Variables	Univariate analysis		Multivariate analysis	
	HR (95% CI)	*P* value	HR (95% CI)	*P* value
Age, years	0.99 (0.98–1.01)	0.211	-	-
Gender (male *vs.* female)	0.98 (0.76–1.28)	0.894	-	-
ECOG (1 *vs.* 0)	1.39 (1.07–1.80)	**0.013**	1.49 (1.14–1.94)	**0.004**
ECOG (2 *vs.* 0)	1.56 (0.64–3.82)	0.332	1.95 (0.79–4.81)	0.148
BMI, kg/m^2^	0.98 (0.94–1.03)	0.473	-	-
Smoking history (yes *vs.* no)	0.94 (0.72–1.22)	0.641	-	-
Drinking history (yes *vs.* no)	0.86 (0.63–1.17)	0.343	-	-
Diabetes history (yes *vs.* no)	1.26 (0.96–1.66)	0.101	-	-
Surgery before chemotherapy (yes *vs.* no)	1.09 (0.77–1.56)	0.619	-	-
Baseline stage (metastatic *vs.* locally advanced)	1.80 (1.37–2.37)	**< 0.001**	1.26 (0.84–1.89)	0.259
Baseline CA19-9 level, U/mL	1.00 (1.00–1.00)	**0.076**	1.00 (1.00–1.00)	0.302
Liver metastases (yes *vs.* no)	1.98 (1.52–2.57)	**< 0.001**	1.78 (1.22–2.59)	**0.003**
Multiple metastases (yes *vs.* no)	1.44 (1.02–2.04)	**0.037**	1.06 (0.73–1.53)	0.758
First-line regimen (GS *vs.* GnP)	0.82 (0.62–1.08)	0.157	-	**-**

The bold values indicate *P* < 0.1 in the univariable analysis and *P* < 0.05 in the multivariable analysis.

### Second- and third-line therapies

A total of 272 patients were included in the later-line chemotherapy study. The use of subsequent antitumor therapy was balanced between treatment groups: 70.7% in the GS group and 61.1% in the GnP group (*P* = 0.162) for second-line chemotherapy, and 34.1% in the GS group and 32.6% in the GnP group (*P* = 0.918) for third-line chemotherapy (**Table 5**). The most common second-line regimen in the GS group was GnP (25.9%), whereas that in the GnP group was S1 plus oxaliplatin (35.3%).

**Table 5:** Second-line and third-line chemotherapy (*n* = 272)

	First-line chemotherapy regimen	
	GS (*n* = 82)	GnP (*n* = 190)
**Overall**		
Second-line	58 (70.7%)	116 (61.1%)
Third-line	28 (34.1%)	62 (32.6%)
Supportive care	12 (14.6%)	40 (21.1%)
Death	3 (3.7%)	6 (3.2%)
Unknown	9 (11.0%)	28 (14.7%)
**Second-line**				
SOX	6 (10.3%)	41 (35.3%)
GS	6 (10.3%)	8 (6.9%)
GnP	15 (25.9%)	8 (6.9%)
Gemcitabine	2 (3.4%)	2 (1.7%)
GEMOX	6 (10.3%)	4 (3.4%)
FOLFIRINOX	0 (0.0%)	23 (19.8%)
FOLFIRI	1 (1.7%)	4 (3.4%)
FOLFOX	0 (0.0%)	3 (2.6%)
S-1	2 (3.4%)	9 (7.8%)
Irinotecan + S-1	0 (0.0%)	2 (1.7%)
XELOX	6 (10.3%)	2 (1.7%)
PARPi	0 (0.0%)	3 (2.6%)
Nab-P	2 (3.4%)	0 (0.0%)
Nab-P + S-1	2 (3.4%)	1 (0.9%)
Nab-P + oxaliplatin	5 (8.6%)	0 (0.0%)
Others	5 (8.6%)	6 (5.2%)

FOLFIRI, irinotecan plus fluorouracil and leucovorin; FOLFOX, oxaliplatin plus fluorouracil and leucovorin; GEMOX, gemcitabine plus oxaliplatin; Nab-P, nab-paclitaxel; PARPi, poly ADP-ribose polymerase inhibitor; SOX, S-1 plus oxaliplatin; XELOX, capecitabine plus oxaliplatin.

The responses to second-line chemotherapy are shown in **Supplementary Table S5**. Among all evaluable patients, 6 (4.7%) achieved partial response, 48 (37.2%) achieved stable disease, and 75 (58.1%) achieved progressive disease. The ORR in the GS group was slightly lower than that in the GnP group (2.4% *vs.* 5.7%; *P* = 0.686). The DCR was similar between groups (40.5% *vs.* 42.5%, *P* = 0.975).

### AEs

Evaluable safety data were available for 245 patients. Grade 3 or 4 AEs were observed in 42 of the 67 (62.7%) patients administered GS and 125 of the 178 (70.2%) patients administered GnP as first-line chemotherapy (**Table 6**). The incidence of leukopenia was significantly lower in the GS group than the GnP group (14.9% *vs.* 28.1%, *P* = 0.049). The grade 3 or 4 AEs frequently (≥ 5%) experienced by the GS group were leukopenia, neutropenia, thrombocytopenia, hyperbilirubinemia, and oral mucositis. Leukopenia, neutropenia, thrombocytopenia, anemia, fatigue, and peripheral neuropathy were frequently observed in the GnP group (< 5%).

**Table 6:** Grade 3 or 4 AEs (*n* = 245)

	First-line chemotherapy regimen		*P* value
	GS (*n* = 67)	GnP (*n* = 178)	
**Overall**			0.329
No	25 (37.3%)	53 (29.8%)	
Yes	42 (62.7%)	125 (70.2%)	
**Hematologic**			
Leukopenia			0.049
No	57 (85.1%)	128 (71.9%)	
Yes	10 (14.9%)	50 (28.1%)	
Neutropenia			0.988
No	35 (52.2%)	95 (53.4%)	
Yes	32 (47.8%)	83 (46.6%)	
Thrombocytopenia			0.386
No	60 (89.6%)	167 (93.8%)	
Yes	7 (10.4%)	11 (6.2%)	
Anemia			0.839
No	64 (95.5%)	167 (93.8%)	
Yes	3 (4.5%)	11 (6.2%)	
**Nonhematologic**			
Fatigue			0.839
No	64 (95.5%)	167 (93.8%)	
Yes	3 (4.5%)	11 (6.2%)	
Nausea			0.614
No	64 (95.5%)	174 (97.8%)	
Yes	3 (4.5%)	4 (2.2%)	
Vomiting			0.896
No	66 (98.5%)	173 (97.2%)	
Yes	1 (1.5%)	5 (2.8%)	
Rash			1.000
No	64 (95.5%)	170 (95.5%)	
Yes	3 (4.5%)	8 (4.5%)	
Diarrhea			0.893
No	65 (97.0%)	175 (98.3%)	
Yes	2 (3.0%)	3 (1.7%)	
Anorexia			1.000
No	64 (95.5%)	170 (95.5%)	
Yes	3 (4.5%)	8 (4.5%)	
Bilirubin			0.085
No	63 (94.0%)	176 (98.9%)	
Yes	4 (6.0%)	2 (1.1%)	
ALT/AST			1.000
No	66 (98.5%)	175 (98.3%)	
Yes	1 (1.5%)	3 (1.7%)	
Hand-foot syndrome			0.611
No	66 (98.5%)	178 (100.0%)	
Yes	1 (1.5%)	0 (0.0%)	
Oral mucositis			0.085
No	63 (94.0%)	176 (98.9%)	
Yes	4 (6.0%)	2 (1.1%)	
Peripheral neuropathy			0.135
No	67 (100.0%)	169 (94.9%)	
Yes	0 (0.0%)	9 (5.1%)	

## Discussion

Gemcitabine has been one of the most used chemotherapeutic drugs for PDAC since its superiority to fluorouracil was reported in 1997^3^. Gemcitabine has been combined with other drugs, such as fluorouracil, platinum, irinotecan, and erlotinib. However, the survival benefits of these combination regimens *vs.* gemcitabine alone are limited and inconsistent^14–20^. Currently, the combination of nab-paclitaxel plus gemcitabine is widely accepted, owing to its superior efficacy to that of gemcitabine alone^5^. However, the higher risk of severe AEs has restricted its use, and its treatment efficacy remains unsatisfactory. Because S-1, which is administered as a single agent for advanced and resected PDAC, is not inferior to gemcitabine and has lower toxicity^7,21^, efforts have been made to develop a new combination comprising S-1 and gemcitabine. GS has been reported to be a potentially better alternative to gemcitabine and has been well-tolerated in several phase II trials^22–25^. Although 4 RCTs comparing GS with gemcitabine alone have shown inconsistent results regarding OS^7,10–12^, a meta-analysis has confirmed the survival benefits of GS as first-line chemotherapy for advanced PDAC^26^.

**Figure 5:**
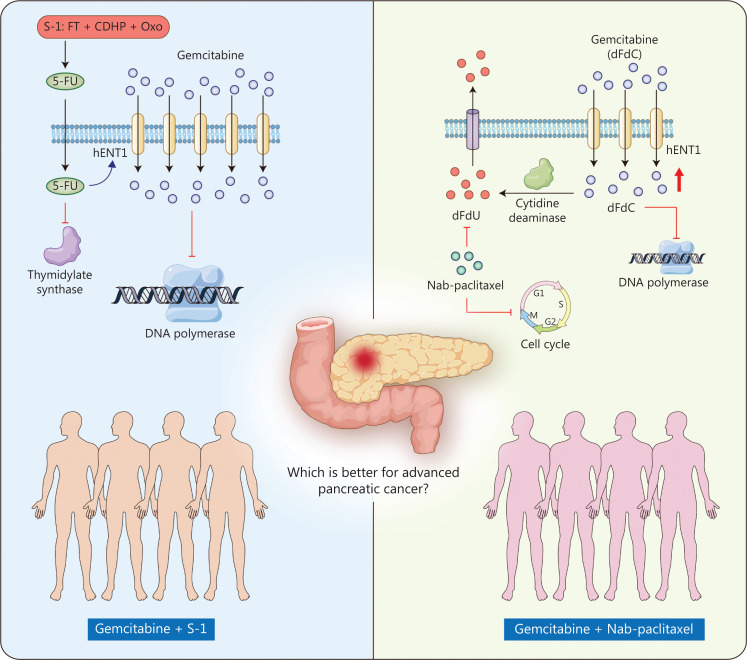
Schematic of the mechanisms of GS and GnP. For GS, S-1 is an oral derivative of the 5-FU prodrug combined with 2 modulators. The expression of hENT1, the mediator of gemcitabine uptake, is upregulated by 5-FU. For GnP, nab-paclitaxel increases gemcitabine levels by inhibiting the gemcitabine-metabolizing enzyme cytidine deaminase. FT, tegafur; CDHP, gimeracil; Oxo, oteracil.

In preclinical studies, GS and GnP have shown synergistic efficacy in murine models of PDAC. The 2 drugs in the combined regimens have different mechanisms of antitumor action and have shown no cross-resistance. However, the underlying mechanisms through which S-1 and nab-paclitaxel increase the efficacy of gemcitabine differ. Human equilibrative nucleoside transporter 1 (hENT1) is a major mediator of gemcitabine uptake. Thymidylate synthase inhibitors, such as 5-FU, upregulate the expression of hENT1. S-1 is an oral derivative of the 5-FU prodrug combined with 2 modulators that maintain effective 5-FU concentrations in plasma and tumor tissues^8^. For GnP, nab-paclitaxel decreases the levels of the primary gemcitabine-metabolizing enzyme, cytidine deaminase, thus increasing intratumoral gemcitabine levels^27^. No preclinical or clinical studies have directly compared the efficacy of GS and GnP. On the basis of the promising efficacy and mechanistic interpretation of the GS regimen, we directly compared GS with GnP as first-line treatment for advanced PDAC (**Figure 5**).

This study comprised 300 patients with advanced PDAC. The median OS statistically significantly improved in the GS group (17.9 months *vs.* 13.3 months; *P* = 0.0078). However, the PFS was comparable between groups. On the basis of multivariate Cox analyses, the choice of first-line chemotherapy regimen (GS or GnP) was associated with OS. The GS regimen was an independent predictor of longer OS in patients with advanced PDAC. However, the regimen choice was not associated with PFS, possibly because of the influence of baseline confounding factors, because both OS and PFS were significantly longer with GS in the propensity-matched cohort. Nevertheless, these results warrant further investigation in a larger prospective cohort.

To better evaluate the efficacy of each chemotherapy regimen, we analyzed later-line therapies. The frequency of subsequent chemotherapy was statistically balanced between treatment groups. Notably, a numerically higher rate of second-line chemotherapy was observed in the GS group than the GnP group (70.7% *vs.* 61.1%, *P* = 0.165). Because more patients received second-line therapy in the GS group, the OS might have been increased. However, both the ORR and DCR of second-line chemotherapy were lower in the GS group than the GnP group. Therefore, as first-line chemotherapy, the GS regimen achieved a longer OS than the GnP regimen.

On the basis of a comparison with previous studies with GS or GnP^5,7,10–12,28,29^, the OS rates of 17.9 months with GS and 13.3 months with GnP in the present study were among the best results for patients with advanced PDAC. The longer OS in both the GS and GnP groups might have been due to the inclusion of 28 (33.3%) patients in the GS group and 83 (38.4%) patients in the GnP group with locally advanced PDAC. In addition, 12 (14.3%) patients with GS and 36 (16.7%) patients with GnP underwent surgery for the primary lesion before first-line chemotherapy, and 8 patients (2 with GS and 6 with GnP) underwent surgery after first-line chemotherapy.

Regarding efficacy, we evaluated not only the radiographic response according to the RECIST guidelines but also the serologic response by measuring changes in CA19-9 levels. On the basis of our results, both the radiographic and serological response metrics were comparable between the GS and GnP groups, even after propensity score matching to control for confounding factors. Notably, the ORR in the GS group (14.3%) was slightly higher than that in the GnP group (9.7%), whereas the DCR was similar between groups (81.0% *vs.* 74.5%). The ORR of GS and GnP in this study was lower than reported in previous trials (17%–29% *vs.* 9%–25%), whereas the DCR was slightly higher than reported in other studies (64%–77% *vs.* 50%–91%)^5,7,10–12,28,29^. This inconsistency might be due to the strict selection criteria and monitoring performed in prospective trials.

On the basis of subgroup analyses, patients receiving GS as first-line chemotherapy had longer OS than those receiving GnP, particularly among male patients; patients with a smoking history; patients with an ECOG performance status score of 0; patients with localized PDAC; and patients without elevated CA19-9 levels, liver metastases, or multiple metastases. In the MPACT trial, subgroup analyses have suggested that patients with poorer performance status, liver metastasis, more than 3 metastatic sites, metastatic PDAC at baseline, or markedly elevated CA19-9 levels have greater survival benefits with first-line GnP than gemcitabine monotherapy^5^. In a randomized phase III study, GS had a better HR than gemcitabine alone in a subgroup of patients with locally advanced PDAC^7^. In our study, we directly compared GS with GnP and confirmed that patients with less advanced PDAC achieved a greater decrease in the risk of death when they received GS as first-line chemotherapy.

The incidence of grade 3 or 4 AEs was lower in the GS group (62.7%) than the GnP group (70.2%). In a phase III RCT, neutropenia of grade 3 or worse was observed in 62.2% of patients in the GS group^7^, in agreement with our results. In addition, the incidence of leukopenia was significantly lower in the GS group, and peripheral neuropathy was frequently observed in the GnP group. Notably, the completion rate of chemotherapy with GS was significantly higher than that with GnP (50.0% *vs.* 30.3%; *P* = 0.0028). The higher completion rate suggests that the GS regimen is better tolerated than GnP as first-line chemotherapy.

In the present study, we reported the real-world outcomes at a single center, comparing GS with GnP as first-line chemotherapy for patients with advanced PDAC. To our knowledge, this is the first study comparing the efficacy and safety for GS *vs.* GnP. However, this study has several limitations. First, this study was retrospective in nature and had inherent selection bias. In addition, the incidence of AEs might have been affected by monitoring bias. Second, only patients from a single center were evaluated. Third, S-1 is widely used in Asia; however, only Chinese patients were assessed in the present study. Of note, the pharmacokinetics and pharmacodynamics of S-1 in might differ between white and East Asian patients^30^. Therefore, the role of S-1 combined with gemcitabine as first-line chemotherapy should be re-assessed in non-Asian patients with PDAC.

## Conclusions

In summary, as first-line chemotherapy, the GS regimen, compared with the GnP regimen, resulted in a significantly longer OS and a lower incidence of leukopenia. PFS and ORR were comparable between treatment groups. Further prospective studies are warranted to determine the optimal first-line chemotherapeutic regimen for patients with advanced PDAC.

## Data Availability

Data were generated by the authors and available on request.
