# Supplementary material for: Efficacy and safety of gemcitabine plus S-1 vs. gemcitabine plus nab-paclitaxel in treatment-naïve advanced pancreatic ductal adenocarcinoma
Source: Cancer Biol Med. 2023 Aug 29;20(10):765–78. doi: 10.20892/j.issn.2095-3941.2023.0189 (PMC10618946; doi:10.20892/j.issn.2095-3941.2023.0189)
Supplement: Supplementary file 1 [file cbm-20-765-s001.pdf]

Supplementary materials

Table S1 Baseline characteristics of the 142 matched patients

	Overall *n* = 142	First-line chemotherapy regimen		*P* value
		GS (*n* = 71)	GnP (*n* = 71)	
Gender				0.606
Male	86 (60.6%)	41 (57.7%)	45 (63.4%)	
Female	56 (39.4%)	30 (42.3%)	26 (36.6%)	
Age, years				0.858
≤ 65	96 (67.6%)	49 (69.0%)	47 (66.2%)	
> 65	46 (32.4%)	22 (31.0%)	24 (33.8%)	
ECOG				0.609
0	84 (59.2%)	44 (62.0%)	40 (56.3%)	
1	58 (40.8%)	27 (38.0%)	31 (43.7%)	
BMI, median (range), kg/m^2	21.8 (15.0, 35.3)	21.9 (15.0, 27.6)	21.6 (16.2, 35.3)	0.950
Smoking history				0.229
Yes	56 (39.4%)	24 (33.8%)	32 (45.1%)	
No	86 (60.6%)	47 (66.2%)	39 (54.9%)	
Drinking history				0.315
Yes	32 (22.5%)	13 (18.3%)	19 (26.8%)	
No	110 (77.5%)	58 (81.7%)	52 (73.2%)	
Diabetes history				0.465
Yes	43 (30.3%)	19 (26.8%)	24 (33.8%)	
No	99 (69.7%)	52 (73.2%)	47 (66.2%)	
Surgery before chemotherapy				0.244
Yes	13 (9.2%)	9 (12.7%)	4 (5.6%)	
No	129 (90.8%)	62 (87.3%)	67 (94.4%)	
Elevated CA19-9				1.000
Yes	113 (79.6%)	57 (80.3%)	56 (78.9%)	
No	29 (20.4%)	14 (19.7%)	15 (21.1%)	
Baseline stage				1.000
Locally advanced	52 (36.6%)	26 (36.6%)	26 (36.6%)	
Metastatic	90 (63.4%)	45 (63.4%)	45 (63.4%)	
Liver metastases				0.737
Yes	69 (48.6%)	33 (46.5%)	36 (50.7%)	
No	73 (51.4%)	38 (53.5%)	35 (49.3%)	
Multiple metastases				0.502
Yes	24 (16.9%)	10 (14.1%)	14 (19.7%)	
No	118 (83.1%)	61 (85.9%)	57 (80.3%)	

Table S2 Response to chemotherapy among the 142 matched patients

	Overall n = 142	First-line chemotherapy regimen		P value
		GS (n = 71)	GnP (n = 71)	
Best response				0.202
CR	0	0	0	
PR	17 (12.0%)	11 (15.5%)	6 (8.5%)	
SD	96 (67.6%)	49 (69.0%)	47 (66.2%)	
PD	29 (20.4%)	11 (15.5%)	18 (25.4%)	
ORR	17 (12.0%)	11 (15.5%)	6 (8.5%)	0.301
DCR	113 (79.6%)	60 (84.5%)	53 (74.6%)	0.212
Change in CA19-9				0.870
Not expressed	28 (19.7%)	14 (19.7%)	14 (19.7%)	
Declined > 30%	82 (57.7%)	42 (59.2%)	40 (56.3%)	
Declined ≤ 30%	26 (18.3%)	13 (18.3%)	13 (18.3%)	
Unknown	6 (4.2%)	2 (2.8%)	4 (5.6%)	

CR, complete response; DCR, disease control rate; ORR, objective response rate; PD, progressive disease; PR, partial response; SD, stable disease.

Table S3 Univariate and multivariate analyses of overall survival among the 142 matched patients

Variables	Univariate analysis		Multivariate analysis	
	HR (95% CI)	P value	HR (95% CI)	P value
Age, years (> 65 *vs.* ≤ 65)	0.91 (0.58–1.41)	0.660	-	-
Gender (male *vs.* female)	1.01 (0.67–1.52)	0.979	-	-
ECOG (1 *vs.* 0)	1.99 (1.32–3.01)	**0.001**	2.58 (1.66–4.01)	**< 0.001**
BMI, kg/m^2	0.98 (0.92–1.05)	0.631	-	-
Smoking history (yes *vs.* no)	0.97 (0.64–1.48)	0.899	-	-
Drinking history (yes *vs.* no)	1.10 (0.69–1.75)	0.691	-	-
Diabetes history (yes *vs.* no)	1.24 (0.80–1.92)	0.343	-	-
Surgery before chemotherapy (yes *vs.* no)	0.87 (0.44–1.75)	0.700	-	-
Baseline stage (metastatic *vs.* locally advanced)	1.74 (1.14–2.67)	**0.001**	0.84 (0.42–1.69)	0.622
Elevated CA19-9 level (yes *vs.* no)	1.31 (0.77–2.25)	0.316	-	-
Liver metastases (yes *vs.* no)	2.26 (1.49–3.42)	**< 0.001**	3.26 (1.63–6.53)	**< 0.001**
Multiple metastases (yes *vs.* no)	1.69 (0.98–2.91)	**0.060**	1.02 (0.56–1.84)	0.951
First-line regimen (GS *vs.* GnP)	0.55 (0.36–0.85)	**0.006**	0.60 (0.39–0.93)	**0.024**

The bold values indicate P < 0.1 in the univariable analysis and P < 0.05 in the multivariable analysis.

Table S4 Univariate and multivariate analyses of progression-free survival among the 142 matched patients

Variables	Univariate analysis		Multivariate analysis	
	HR (95% CI)	*P* value	HR (95% CI)	*P* value
Age, years (> 65 *vs.* ≤ 65)	1.11 (0.76–1.63)	0.592	-	-
Gender (male *vs.* female)	0.91 (0.63–1.30)	0.593	-	-
ECOG (1 *vs.* 0)	1.56 (1.08–2.24)	**0.018**	1.86 (1.28–2.70)	**0.001**
BMI, kg/m^2	0.98 (0.93–1.04)	0.550	-	-
Smoking history (yes *vs.* no)	0.83 (0.57–1.20)	0.314	-	-
Drinking history (yes *vs.* no)	0.77 (0.50–1.19)	0.246	-	-
Diabetes history (yes *vs.* no)	1.01 (0.68–1.49)	0.978	-	-
Surgery before chemotherapy (yes *vs.* no)	1.17 (0.64–2.13)	0.615	-	-
Baseline stage (metastatic *vs.* locally advanced)	1.92 (1.32–2.81)	**0.001**	1.43 (0.83–2.46)	0.193
Elevated CA19-9 level (yes *vs.* no)	1.47 (0.93–2.33)	**0.098**	1.68 (1.05–2.70)	**0.031**
Liver metastases (yes *vs.* no)	1.99 (1.37–2.88)	**< 0.001**	1.84 (1.09–3.13)	**0.023**
Multiple metastases (yes *vs.* no)	1.50 (0.92–2.47)	0.106	-	-
First-line regimen (GS *vs.* GnP)	0.55 (0.38–0.80)	**0.002**	0.51 (0.35–0.75)	**0.001**

The bold values indicate $P < 0.1$ in the univariable analysis and $P < 0.05$ in the multivariable analysis.

Table S5 Response to second-line chemotherapy (n = 129)

	Overall n = 129	First-line chemotherapy regimen		*P* value
		GS (n = 42)	GnP (n = 87)	
Best response to second-line chemotherapy				0.696
CR	0	0	0	
PR	6 (4.7%)	1 (2.4%)	5 (5.7%)	
SD	48 (37.2%)	16 (38.1%)	32 (36.8%)	
PD	75 (58.1%)	25 (59.5%)	50 (57.5%)	
ORR	6 (4.7%)	1 (2.4%)	5 (5.7%)	0.686
DCR	54 (41.9%)	17 (40.5%)	37 (42.5%)	0.975

CR, complete response; DCR, disease control rate; ORR, objective response rate; PD, progressive disease; PR, partial response; SD, stable disease.
